# The Impact of Health Insurance on Low Birth-Weight Infants and Mothers at a Tertiary Care Hospital in Tabuk, Saudi Arabia: A Retrospective Case-Control Study

**DOI:** 10.7759/cureus.31000

**Published:** 2022-11-02

**Authors:** Sajjan Iqbal Memon, Kiran Afzal, Aamir Gul Memon, Noor us Saba Shaikh, Umair Ismail Manghrio

**Affiliations:** 1 Department of Health Administration, King Saud University, Riyadh, SAU; 2 Department of Physical Therapy, Comwave Institute, Islamabad, PAK; 3 Department of Physical Therapy, Riphah International University, Lahore Campus, Lahore, PAK; 4 Department of Physical Therapy, Al-Biruni Institute of Physical Therapy and Rehabilitation Sciences, Hyderabad, PAK; 5 Department of Molecular Medicine and Pathology, King Salman Armed Forces Hospital, Tabuk, SAU

**Keywords:** saudi arabia, females, infants, health insurance, low birth weight

## Abstract

Objectives: To determine the impact of having private health insurance during the period of maternity on low birth-weight (LBW) infants.

Methods: This retrospective case-control study was carried out at a tertiary care hospital in Tabuk, Saudi Arabia, between January 2020 and January 2021. Using non-probability sampling, secondary medical data were obtained at the Department of Obstetrics & Gynecology from two groups: insured and non-insured mothers who had 150 LBW infants (LBWI) (<2.5 kg) as well as normal newborns. Data were analyzed using SPSS, version 24 (IBM Corp., Armonk, NY). A univariate analysis was performed for each variable followed by a logistic regression analysis to explain the relationship between the dependent (LBW) and independent variables (health insurance status, prenatal care, and inter-pregnancy interval).

Results: Out of 300 mothers, the majority were in the age group 21-34 years (86%). The LBWI mothers were insured in about 55% of cases. Around 43% had insurance for 1-2 years, and 44.7% of insured mothers were covered by a “class A” health plan. The findings revealed a significant correlation between the duration of maternal insurance coverage period and LBW; it reduced the risk of LBW by 95% CI. The inter-pregnancy interval was 51.3%, with a p-value of 0.33.

Conclusion: Private health insurance offers coverage, stability, and consistency in Saudi Arabia, which results in better birth outcomes by decreasing both infant mortality and morbidity rates among insured females.

## Introduction

The birth weight of an infant is regarded as the first weight recorded immediately after delivery, which is measured within a few hours before any significant postnatal weight loss, and the World Health Organization (WHO) considers birth weight below 2.5 kg as low birth weight (LBW); 20% of all newborns are considered LBW, a life-threatening risk among infants around the globe, and the infants born to medically insured mothers are likely to have a normal birth weight [1]. Moreover, the consequences of LBW are substantial on both infants and mothers, as they are associated with neurodevelopmental impairment, family stress, and significant societal costs [2]. LBW infants (LBWI) have medical and social complications and concluded that LBW is positively associated with neonatal morbidity and disability, with a 48% higher risk of death than normal-weight infants during their neonatal period [3-5].

The comprehensive medical package, which covers a wide range of medical services, has been found to reduce the newborn and maternal mortality risk, thereby increasing access to infant and maternal health services [6,7]. In addition, neurosensory disability drives a major complication associated with LBW; it can reduce the incidence with greater access to antenatal corticosteroids and neonatal intensive care [8].

As health insurance is aimed at protecting patients after a risky event, maternal insurance coverage reduces financial obstacles to health care services and improves health outcomes [8-10]. Hypertension appears to be more prevalent among adults who were born LBWIs, with an inverse correlation between systolic blood pressure and birth weight [11]. The LBWIs have a much higher mortality rate and are often associated with neurologic disability, impaired education development, and chronic diseases such as cardiovascular disease [12,13]. However, the Saudi government and community are well aware of the consequences of LWB in the long run and the need for preventive measures [14]. Various empirical studies have found that maternal and newborn healthiness are major predictors of a community’s wellness [15-17]. These studies have contended that easy access to high-quality prenatal care is the primary contributor to improving maternal facilities and reducing the risk of LBWIs [5].

Long-term new initiatives were implemented by the Saudi government to provide better healthcare services to the Saudi community through subsidized insurance policies. An extensive literature has revealed that the affluent, upper-middle, and lower-middle classes in the Saudi community are well aware and inclined toward comprehensive insurance coverage to avoid unpleasant situations in the quality of health care, particularly during the time frame of maternal and newborn quality of health care [16]. Considering these studies and some other unpublished reports, the Saudi government has prioritized maternal facilities and the risk of LBW. This realization is clear among regulatory bodies but is taking a long time to implement because of other issues in the national health care system, which also need to be addressed. Therefore, relying on the mentioned premise, this study attempts to determine the effect of health-insured mothers on LBWIs in Saudi society.

The objective of this study was to accomplish various methodological steps drawn from evidence based on logical inferences about the impact of health insurance on LBW among insured and uninsured mothers.

## Materials and methods

Study design

Between January 2020 and January 2021, a retrospective case-control study was carried out at a 470-bed tertiary care hospital in Tabuk, Saudi Arabia. This is an observational study in which two groups of the target population already exist in the form of insured and uninsured mothers of LBWIs. The infants’ weights were measured using a digital calibrated scale. Insurance coverage data were obtained from the hospital’s finance and admissions departments. The Ethics Committee Review Board of King Salman Armed Forces Hospital, Tabuk issued approval for the study (IRB-KSAFH-0036/4/20).

Population and sampling 

The study population consists of LBW infant cases recorded by a tertiary private hospital. Multiple births and stillbirths were excluded. The sample frame was a list of LBWIs’ mothers who gave birth to newborn babies during the set period. Furthermore, non-Saudi mothers were also excluded to control for any heterogeneity effect. The sample unit was a single LWBI, and its size consist of 300 mothers with their newborn babies. The non-probability sampling technique was used to select the sample units based on medical opinion and to illustrate the singularity of LWBIs under investigation. The sample included 150 mothers with LBWIs. All subjects were selected based on where they were living for the previous five years. Only subjects who were private and insured were recruited.

The fieldwork was conducted and medical records of the study participants were accessed at the hospital following deliveries throughout the specified time period. The group-matching analysis was performed based on newborn infants’ gender, delivery type, and maternal age. A total of 150 LBWIs cases and 150 controls were classified to compare the outcomes of participants. The sampling adequacy of this study was estimated using the Lwanga and Lemeshow table for a 95% CI.

Measurement and procedure

The data were collected from the medical records of private health-insured mothers of LBWI cases in the maternity wards of private hospitals. The dependent (LBW infant cases) and independent (health insurance status; prenatal care status; inter-pregnancy interval) variables of the study were measured through their critical properties by using a checklist that consisted of 212 items. The critical items of each variable were identified from an extensive review of the literature that has been used in various contexts [18]. 

The checklist included nine items related to risk factors associated with LBWIs cases; three were associated with gestational age, weight, and inter-pregnancy interval; four items were linked to health insurance status; and two were related to residential area and prenatal care status. The prenatal care status comprised three items that were extracted from Kessner Index [19]. These items were modified according to the objectives of this study. The modifications were slight and linguistic. The prenatal care status was measured adequately before or during the third month of gestation, which included five or more visits. It was considered insufficient if it began during or after the seventh month of gestation with four or fewer prenatal visits. The prenatal care status that did not meet the measurement criteria of adequate or inadequate care was classified as intermediate. The inter-pregnancy interval was calculated as the period from the last previous birth to the start of the current pregnancy.

The raters were based on their experiences and conversant about the LBWIs and their consequences. The physicians, research assistants, and head nurses were contacted personally to explain the specific aims of the study and to assure the confidentiality of the collected information. The operational definitions of study variables and their critical items were provided on the first page of the checklist to avoid any kind of misinterpretation of the given statement. The set criteria and detailed guidelines were also provided about the inclusion and exclusion of required data. The collected data were validated in the internal health information system, which depends on match-cases found in medical records.

Health insurance-coverage status was tested using two items: degree and duration of coverage. The degree of coverage was divided into five classes of insurance plans practised in Saudi Arabia’s health insurance schemes. The length of coverage before birth was used to calculate the period of coverage. Participants with less than one year of insurance coverage before delivery were excluded. Maternal age was further classified into three categories: 20 years or less, 21-34 years, and over 35 years. The region of residence was divided into two categories: rural and urban. Moreover, prenatal care was also divided into three categories: moderate, inadequate, and adequate.

Statistical analysis 

The adjusted strata multiple regression statistical technique was used to analyze the collected data to predict the significance levels of the dependent/independent variables of this study. The Statistical Package for the Social Sciences (SPSS) for Windows, version 24.0 (IBM Corp., Armonk, NY) was used to run the adjusted strata multiple regression test. The odds ratios were obtained at 95% CI by categorizing continuous variables such as maternal age, weight gain, and health insurance duration in terms of discrete forms. In this study, the categories were compared to the reference based on the literature to categorize the risk factors associated with LBWIs.

A univariate analysis was initially performed for each variable. Then, logistic regression analysis was conducted to explain the relationship between a dependent (LBW) and independent variable (health insurance status; prenatal care status; inter-pregnancy interval). The independent variables were found to be significant (p < 0.05), and odds ratios greater than one was entered into the stepwise multinomial logistic regression. Multinomial logistic regression was used to generalize the logistic regression to multiclass glitches and to understand the relationship between LBWIs as the dependent variable (0 = LBWI case and 1 = control), while the insurance status and LBWI risk factors were categorized as independent variables. The results were reported as odds ratios (95% CI). The odds ratio was calculated by obtaining the odds ratio and relative risk at a 95% CI and from the regression coefficient and its standard error. A goodness of fit for the regression model was also tested.

## Results

A total of 10,668 births took place throughout the study period, out of which 150 newborns were full-term LBWI. This means there was an incidence of 1.4% or 14 out of every 1000 newborns who were LBWIs. About 37% of the mothers of LBWI were uninsured, whereas 40% had private insurance. 

Table 1 indicates that most mothers were in the age group 21-34 (86%), and 7% were less than 21 or over 34 years. Approximately 55% of the mothers who gave birth to LBWI were insured. Around 43% had insurance coverage for 1-2 years, 8% had coverage for three years, and 5% had coverage for four or more years. The majority of insured mothers were in a “class A” coverage insurance plan: 44.7% and 72.7% of LBWIs cases and normal controls, respectively. Using a univariate analysis, each variable was entered into the logistic regression analysis as the only independent variable, and odds ratios were obtained.

**Table 1:** Maternal Socioeconomic Risk Factors of LBWIs and the Results of Univariate Analysis LBWI: low birth-weight infant.

Factor	Cases	Controls	OR (95%CI)	P-value
	No. (%)	No. (%)		
Insurance Status	1 (Ref)			
Insured	83 (55.3)	125 (83.3)	1 (Ref)	
Not insured	67 (44.7)	25 (16.7)	.248 (.145 - .424)	.000
Insurance class of coverage				
Highly Important Patient (VVIP)	1 (.7)	2 (1.3)	1 (Ref)	.178
Very Important Patient	7 (4.7)	7 (4.7)	.187 (.0162 - .149)	.091
network coverage plan (Class A)	67 (44.7)	109 (72.7)	.373 (.1191 - .171)	.000
network coverage plan (Class B)	8 (5.3)	7 (4.7)	.229 (.132 - .398)	.134
Duration of last insurance				
1<3	6 (42.7)	36 (24)	-1.707 (1.707 - 1.707)	.225
3	12 (8)	35 (23.3)	-2.056 (.368 - 1.265)	.000
3<	7 (4.7)	54 (36)	-3.029 (.057 - 1.285)	.000
Smoking				
Smoker	26(17.3)	7(4.7)	1 (Ref)	
Non smoker	124(82.7)	143(95.3)	4.283 (1.797 -10.209)	0.001
n= 300, Ref. = Reference Category, * (P<0.05), CI= Confidence Interval				

Table 2 lists a significant association between reduced LBWI cases and the duration of the maternal insurance coverage, as indicated by the odds ratio and CI (CI of 95%). Moreover, other significant factors associated with the increased risk of LBWIs included maternal weight gain of less than 9 kg during pregnancy, an inter-pregnancy interval of lesser than three years following previous LBWIs, and preeclampsia.

**Table 2:** Maternal Biological Risk Factors of LBWI and the Results of Univariate Analysis LBWI: low birth-weight infant.

Factor	Cases	Controls	OR (95%CI)	P-value
	No. (%)	No. (%)		
Maternal weight prior to pregnancy				
50>	28 (18.7)	10 (6.7)	3.204 (1.476 - 6.959)	.003
50	32 (21.3)	37 (24.7)	.990 (.570 -1.718)	.350
50<	90 (60)	103 (68.7)	1 (Ref)	.003
Weight gain during pregnancy				
9<	50 (33.3)	136 (90.7)	1 (Ref)	
9>	100 (66.7)	14 (9.3)	19.429 (10.179 - 37.084)	.971
Parity				
1	55 (36.7)	30 (20)	2.188 (1.140 - 4.202)	.000
2	32 (21.3)	56 (37.3)	.682 (.358 - 1.300)	.019
4+	32 (21.3)	27 (18)	1.415 (.702 - 2.849)	.245
3	31 (20.7)	37 (24)	1(Ref)	
Inter-pregnancy interval				
Less than 3	77 (51.3)	41 (27.3)	2.804 (1.733 - 4.537)	.332
3+	73 (48.7)	109 (72.7)	1 (Ref)	
History of LBWI				
Yes	32 (21.3)	4 (2.7)	9.898 (3.404- 28.781)	.000
No	118(78.7)	146(97.3)	1(Ref)	
Preeclampsia				
Yes	81(54)	11(7.3)	14.834(7.420 - 29.657)	.000
No	69(46)	139 (92.7)	1(Ref)	
n= 300, Ref. = Reference Category, * (P<0.05), CI= Confidence Interval				

Table 3 summarizes the results of stepwise multinomial adjusted logistic regression tests. These findings revealed a significant negative association between LBWIs and the duration of health insurance coverage. As the coverage duration increases, the risk of LBWIs decreases. Also, no significant associations were detected for the different classes of insurance coverage.

**Table 3:** Results of Stepwise Multiple Logistic Regressions for Maternal Insurance and LBWI Birth LBWI: low birth-weight infant.

Indicator	B. Coefficient	OR (95%CI)	P-value
Health insurance duration of ≥ 3 years	-15.517	9.5134.096 (2.662-34.000)	.001
Smoking	1.41	5 (1.071- 15.66)	.000
Weight gain during pregnancy < 9 kg	2.557	12.865 (5.492-30.138)	.000
Inter-pregnancy interval of < 3 years	0.93	2.554 (1.151 - 5.664	.000
History of LBWI	2.854	17.361 (4.020- 74.986)	.000
Preeclampsia	2.249	9.480 (3.757-23.924)	.000

## Discussion

We endeavored to identify the impact of private health-insured mothers during the period of maternity on LBWIs in the context of Saudi society. The empirical findings of this study revealed that mothers with private insurance coverage for three years or more were found to be associated with a reduction in the risk of having LBWI by 9%. This result is consistent with the reported findings in the studies by Stenberg et al. [15]. However, a previous study conducted by Ibrahim and O'Keefe revealed no significant association between private health-insured mothers and LBWIs [8]. 

Sociodemographic characteristics play a vital role in infant health outcomes. As a result, the financing of healthcare services has become a major challenge in emerged and emerging economies, particularly for women and children in developing countries having limited access to primary healthcare [2]. The health economics researchers have attempted to investigate the impact of health insurance on the duration of the maternal insurance coverage period. Such findings can enlighten health policymakers and develop some awareness among society at large [1-2].

The LBW rate around the world is inconsistent because of regional variations and prevailing conditions. It is observed from the review of relevant literature in developing countries that the LBWI rate is low in East Asia Pacific, which is approximately 6%, in South Asia around 28%, and in sub-Saharan Africa 13% [13]. In developed countries, such as the United Kingdom, high-income and economically stable regions with a higher standard of living have normal LBWI rates compared to that of developing countries; public healthcare policymakers may have to assimilate the occurrence of LWBIs because it reflects a vital predictor of survival [14,15]. 

This study revealed several biological risk factors that are significantly associated with LBWIs. These constructs may include an inter-pregnancy interval of more than three years, weight gain of less than 9 kg during pregnancy, a history of previous LBWIs, preeclampsia, and maternal smoking. These findings are supported by previous studies that were also conducted in the Saudi context [20,21].

The trends in socioeconomic and political systems are demanding paradigm shifts in every sector of the economy, especially in developing countries. Every government in a developing nation attempts to restructure its institutional practices, including the healthcare sector, to deal with emerging tendencies around the world [3]. The obvious purpose of this restructuring is to minimize the reliance on government funding and that health-related service providers’ organizations are supposed to generate their revenue to meet the demands of society [16]. This paradigm shift may result in some inconsistencies because of the privatization of health care services, particularly during the period of maternity and LBWIs. As no clear association was detected among different classes of insurance coverage, it is likely because most of the sample was in the “A” coverage class (44.7%). In contrast, many of the studies conducted in this domain are based on anecdotal evidence; however, this study is firmly empirical in approach. In this sense, there is a lack of information about the impact of private health-insured mothers and the prevention of LBWIs in the long run. More comfortable access to prenatal care and better service quality, particularly the provision of free supplemental vitamins, could explain the association between a longer duration of private coverage and a lowered risk of an LBWI birth. We attempted to help in filling this knowledge gap by providing additional information that might be of interest to researchers and others in this area.

Like other empirical studies, there are some limitations, including a focus on one group of health insurance classes, as most of the participants were insured in the high class. This study was also limited to public and private sector hospitals in a particular region of Saudi Arabia. Family income was not included in the investigation, even though it is a sociodemographic risk factor associated with LBWIs. Numerous factors can contribute to LBWIs yet; policymakers may consider prenatal private health insurance coverage as one of the significant strategies to minimize the LBWI rate by enforcing national health insurance.

## Conclusions

Having private health insurance may improve access to quality prenatal care as well as maternal satisfaction. The findings of this study could be utilized to improve maternal and child health policy, especially concerning health facilities, antenatal services, and nutritional guidelines, to enhance childbirth outcomes. Saudi Arabia’s stable and consistent private health insurance coverage may result in better birth outcomes, decreasing both infant mortality and morbidity rates.
